# Evolution of human respiratory virus epidemics

**DOI:** 10.12688/f1000research.53392.1

**Published:** 2021-06-04

**Authors:** Nash Rochman, Yuri Wolf, Eugene V. Koonin

**Affiliations:** 1 National Center for Biotechnology Information, National Institutes of Health, Bethesda, MD, 20894, USA

**Keywords:** SARS-CoV2; influenza virus; smallpox virus; epidemic modeling; case fatality ratio

## Abstract

**Background:** It is often assumed that pathogens evolve towards reduced virulence, but counterexamples abound. Faced with a new pathogen, such as SARS-CoV-2, it is highly desirable to be able to forecast the case fatality rate (CFR) and overall disease burden into the future. Considerable effort has been invested towards the development of a mathematical framework for predicting virulence evolution. Although many approaches accurately recapitulate complex outcomes, most rely on an assumed trade-off between CFR and infection rate. It is often impractical to empirically validate this constraint for human pathogens.

**Methods:** A compartment model with parameters tuning the degree to which symptomatic individuals are isolated and the duration of immunity is constructed and evaluated at both short timescales and at equilibrium (when it exists).

**Results:** We reveal kinetic constraints where the variation of multiple parameters in concert leads to decreased CFR and increased pathogen fitness, whereas independent variation of the parameters decreases pathogen fitness. Smallpox, SARS-CoV-2, and influenza are analyzed as diverse representatives of human respiratory viruses. We show that highly virulent viruses, such as smallpox, are likely often constrained by host behavior, whereas moderately virulent viruses, such as SARS-CoV-2, appear to be typically constrained by the relationship between the duration of immunity and CFR.

**Conclusions:** Evolution of human respiratory epidemics appears to be often kinetically constrained and a reduction in CFR should not be assumed. Our findings imply that, without continued public health intervention, SARS-CoV-2 is likely to continue presenting a substantial disease burden. The existence of a parameter regime admitting endemic equilibrium suggests that herd immunity is unachievable. However, we demonstrate that even partial isolation of symptomatic individuals can have a major effect not only by reducing the number of fatalities in the short term but also by potentially changing the evolutionary trajectory of the virus towards reduced CFR.

## Background

The case fatality rate (CFR) and infection rate largely determine the survival of both a pathogen and its host. Constraints imposed by the host behavior and pathogen biology prevent independent variation of these rates. Therefore, trends in virulence evolution can be predicted only through understanding these constraints. Although comprehensive models of the evolution of virulence capable of describing complex environments have been developed, ^1^^–^^5^ most studies to date impose constraints assumed from first principles and lacking experimental or empirical validation. ^6^^,^^7^ Most commonly, a trade-off function is assumed ^8^^,^^9^ between the infection rate and the CFR. It is important to note that different definitions of virulence can lead to dramatically different predictions. ^10^ In this work, we are primarily concerned with long-term trends and consider the CFR exclusively (not the rate of instantaneous mortality or other measures of virulence).

Individual hosts harboring high numbers of pathogen are more likely to die than those harboring lower numbers, but they also shed pathogen at increased rates and therefore could infect a greater number of new hosts. ^11^ However, this straightforward picture is complicated by a landscape of opposite evolutionary outcomes. ^12^ For example, vaccines that alleviate symptoms but still admit infection have been suggested to have induced selective pressures which favor the evolution of strains with increased virulence in malaria ^13^^,^^14^ and decreased virulence for diphtheria and pertussis, ^15^ likely due to differences in the cost of toxin production. ^16^ This demonstrates how predictions of specific outcomes useful for informing public health intervention cannot be generalized across pathogens. The infectivity–virulence trade-off has been demonstrated for malaria. ^14^ Although this could be true more generally, the existing data are scarce, and demonstrating this trade-off for most human pathogens is extremely difficult, due to the impracticality of comprehensive contact tracing. Many strategies to replace the explicit use of this trade-off have been discussed. ^11^ One approach is to construct realistic models of within-host pathogen dynamics. ^17^^,^^18^ This approach provides the ability to make nuanced predictions about specific pathogens and environmental conditions, but requires a deep understanding of the pathogen biology, which is often lacking, especially in the case of newly emergent pathogens. Thus, general models that avoid the use of this constraint as a parameter and without explicit descriptions of within-host pathogen dynamics continue to prove useful.

Towards this goal, we explore a range of epidemiological outcomes for human pathogens modelled using available data on well-characterized respiratory viruses. Our principal aim is to chart a “global phase space”, indicating where endemic equilibrium is possible and to establish the bounds of potential evolutionary trajectories within this phase space without leaving that endemic equilibrium. We do not, however, explicitly conduct an invasion analysis. The ability of an emergent strain to invade depends on many factors that are not considered in the present model and the existence of a path within the global phase space does not guarantee that invasion along that path is possible or probable for any particular virus. Conversely, if a path in the global phase space leads out of endemic equilibrium, this suggests that invasion along that path is impossible. Through this approach, we are able to identify broad evolutionary constraints on pathogen evolution.

The apparent inverse relationship between the time during which the host is asymptomatic but infectious and the CFR, ^19^^–^^27^ as well as the difficulty of vaccination against lower-CFR viruses, such as influenza, relative to higher-CFR viruses, such as smallpox, indeed imply the existence of broad, intrinsic constraints that could link the duration of immunity and CFR. We demonstrate that, in addition to trade-offs, the evolution of CFR is often subject to “kinetic constraints” that prevent CFR reduction by imposing a barrier to increasing the total number of infected hosts, our measure of pathogen fitness. This is analogous to an energetically favorable chemical reaction with a high activation energy.

Consider a simple, reversible chemical reaction between reactant, R, and product, P. When sufficient energy, A1, is applied, R is converted to P. This is the activation energy of the forward reaction. Now consider that P is much more stable than R so that P cannot be converted back to R unless 10×A1 energy is applied. This is the activation energy of the reverse reaction. Given sufficient energy and time, the system will reach an equilibrium in which the quantity of P is 10-fold higher than the quantity of R. However, if insufficient energy is applied, less than A1, the system will remain almost wholly composed of R. Under these conditions, the reaction is kinetically constrained. Now suppose that P is much less stable than R so that P is converted back to R whenever 0.1×A1 energy is applied. In this case, even given sufficient energy and time, the system will remain in a state in which the amount of R is at least 10-fold greater than the amount of P. Under these conditions, the (forward) reaction is thermodynamically constrained.

The evolution of pathogen life history traits may be described as a system of reversible reactions where the “stability” of each set of life history traits is estimated by the total number of infected hosts at endemic equilibrium. The evolution of decreased CFR would be thermodynamically constrained if it were impossible to both increase the total number of infected hosts and decrease CFR. Additionally, the evolution of decreased CFR could be kinetically constrained by many barriers to invasion, which might vary among pathogens and even among host populations for the same pathogen. We sought to identify global kinetic constraints for CFR evolution, where there exists a parameter regime, in which the total number of infected hosts is higher and CFR is lower, but accessing this regime requires simultaneously modifying multiple parameters, some of which might be determined by host behavior. In other words, there may be an evolutionary path to decreased CFR, but it is narrow, even without the consideration of specific barriers to invasion. We show that, for high-CFR viruses such as smallpox, the relationship between the infection rate and CFR is likely a kinetic constraint, rather than an actual trade-off, whereas for intermediate-CFR viruses, such as SARS-CoV-2, there is a kinetic constraint between immunity and CFR. Analysis of such constraints could open avenues for prediction of epidemic outcomes and quantitative validation of such predictions.

## Methods

**Figure 1.**
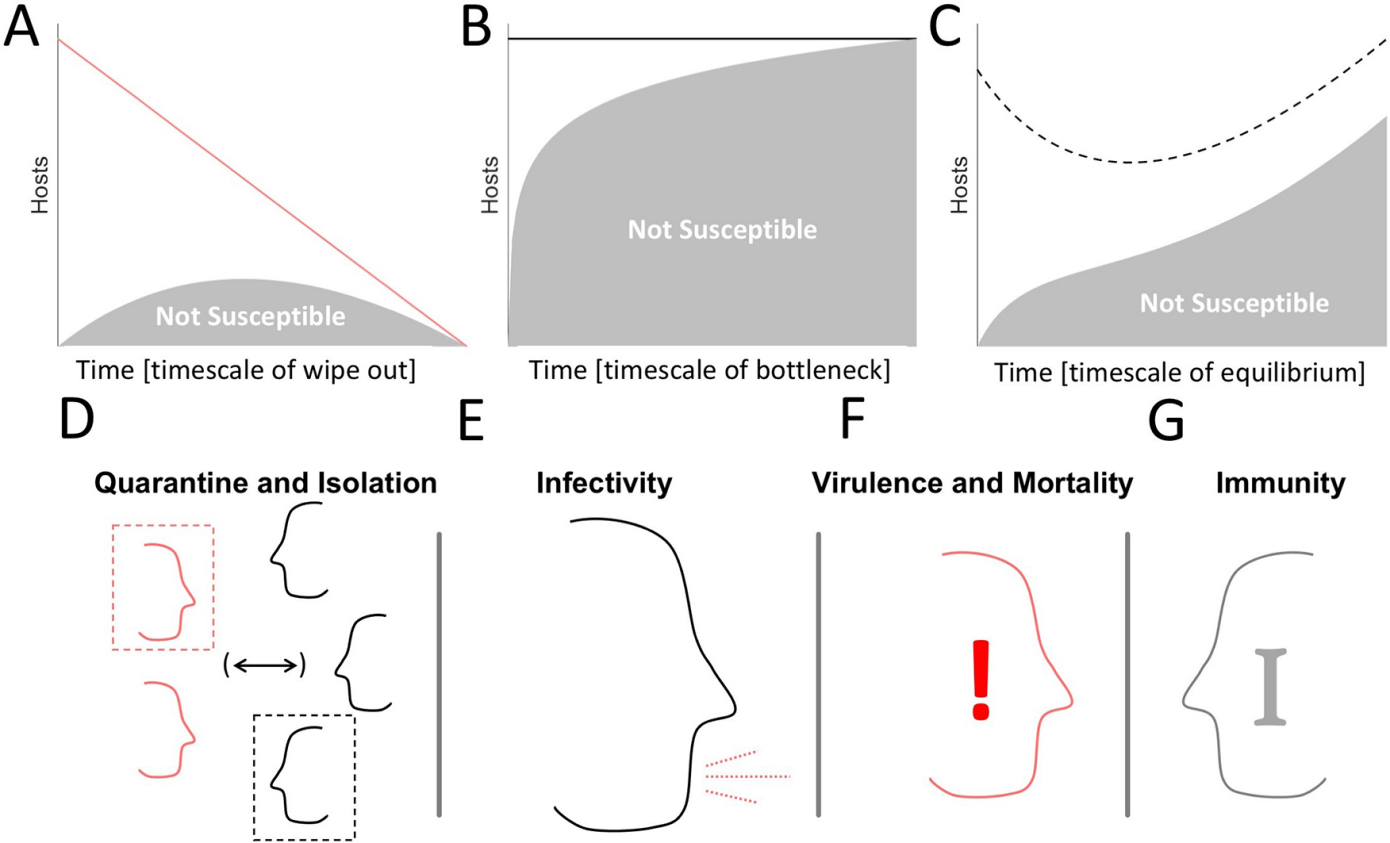
Key factors which determine epidemiological outcomes. A-C. Cartoons representing the three potential outcomes for an epidemic beyond trivial disease-free equilibrium. Lines indicate total number of hosts, shaded areas indicate the fraction of hosts which are not susceptible. A. Host extinction. B. A bottleneck in the susceptible population. C. A balance between infections, CFR, and birthrate. D. Host interaction, quarantine, and isolation. E. Probability of Infection Following Host Contact (Infectivity). F. CFR. G. Immunity.

The introduction of a novel pathogen into a host population is likely to result in one of three outcomes. 1) The number of fatalities is large enough for the pathogen to wipe out the host population (as a result, the pathogen itself also goes extinct) ( Figure 1A). 2) The number of infections is large enough, whereas the number of fatalities is small enough, such that the pathogen creates a bottleneck in the susceptible host population. With all hosts either infected or immune, the pathogen is eliminated from the host population ( Figure 1B). 3) The number of infections is small enough such that a bottleneck in the susceptible population is avoided and long-term persistence within the host population is possible if the number of infections is not too small and the basic reproduction number is greater than one, *R*_0_ > 1 ( Figure 1C). If the basic reproduction number is less than one, disease-free equilibrium is achieved. These three outcomes can occur on vastly different timescales, with a host wipeout or susceptible bottleneck occurring much faster than the rate at which equilibrium is approached. Throughout this work, we simulate each epidemic until one of these three outcomes is reached. With *R*_0_ > 1, a state of stable endemic equilibrium can be reached where the fraction of the host population susceptible to infection remains constant (up to fluctuations). However, if the pathogen-associated fatality exceeds the birth-rate of the host population, the host–pathogen relationship becomes unsustainable in the long term. Such an unsustainable relationship would pose an intense selective pressure on the host population, likely resulting in the extinction of the pathogen through the modification of host behavior or the emergence of resistant hosts.

**Table 1.** Model parameters and key definitions.

α: Probability (permanent) immunity is conferred after infection.
β: Fraction of the symptomatic population which is well mixed (not isolated).
I/i/i*: # of immune hosts/ fraction/fraction at endemic equilibrium
S/s/s*: # of susceptible hosts/ fraction/fraction at endemic equilibrium
A/a/a*: # of asymptomatic and infectious hosts/ fraction/fraction at endemic equilibrium
C/c/c*: # of symptomatic and infectious hosts/ fraction/fraction at endemic equilibrium
k _B_: Birthrate
k _D_: Baseline deathrate unrelated to infection.
k _contact_: Rate of host-host contact
P (infect): Probability of infection given contact between a susceptible and an infected host
k _I_: k _contact_P (infect), the infection rate.
k _P_: Rate of progression from asymptomatic to symptomatic.
k _R_: Rate of recovery from infection.
k _DV_: Rate of death due to infection.
k _L_: (1- α)k _R_(A+C)/I (the rate at which immunity is lost, may be used at endemic equilibrium)
*: Notation indicates reference to endemic equilibrium.
Disease Free Equilibrium: No pathogen remains in the host population (pathogen extinction)
Endemic Equilibrium: A stable fraction of the host population remains infected at large times.
Host Population Crisis: Less than 10% of the initial host population remains.
Susceptible Bottleneck: Less than 10% of the population is susceptible to infection.
Unsustainable: The host population size decreases at endemic equilibrium.
CFR: Case fatality rate.
Fitness: In the context of this work, the total number of infected hosts.
Kinetically Constrained: A regime exists in the parameter space where the total number of infected hosts is larger and the CFR is lower than in the observed regime; however, any evolutionary trajectory that is available to reach that superior domain of the parameter space requires the perturbation of multiple parameters.
Thermodynamically Constrained: No parameter regime exists where the total number of infected hosts is larger and the CFR is lower than in the observed regime.

Which of these courses an epidemic follows, largely depends on the balance of four factors. 1) The frequency of host–host interaction, with or without isolation of infected individuals or prophylactic quarantine ( Figure 1D). 2) The likelihood of an uninfected host to become infected after interacting with an infected host ( Figure 1E). 3) The CFR of the pathogen, that is, the likelihood that an infected host will die as a result of infection ( Figure 1F). 4) The duration of host immunity post infection ( Figure 1G). Although the effect of tuning each of these parameters often appears obvious, for example, decreasing the frequency of host-host interaction almost always decreases the number of infections - many counterintuitive observations become apparent. For example, decreasing the frequency of host-host interaction under conditions that would otherwise lead to a bottleneck in the susceptible population ( Figure 1B) can result in long term persistence within the host population which, avoiding pathogen extinction, ultimately increases the total number of infections sustained over time. In an effort to better delineate how the host-pathogen relationship varies across the space of these factors, we constructed the following model.

Hosts are assigned one of the four possible states: 1) immune, *I*; 2) susceptible, *S*; 3) asymptomatic, *A*; and 4) symptomatic or “clinical”, *C.* New hosts are assumed to be born susceptible at a rate, *k _B_*, and a baseline death rate, *k _D_*, is assumed constant across all compartments. Susceptible hosts can be infected by coming into contact with either asymptomatic or clinical hosts. Asymptomatic hosts either recover at rate, *k _R_*, or progress to the clinical compartment at rate, *k _P_.* Clinical hosts either recover at rate, *k _R_*, or die due to the pathogen at rate, *k _DV_.*

In the model, as written, recovery confers permanent immunity with probability, α. This is done as a mathematical convenience. Under many if not most circumstances, it is more realistic to assume recovery confers immunity which is gradually lost at a constant rate, $k_{L}$. At endemic equilibrium, one may solve for the parameter $k_{L}=\left(1-\alpha \right)k_{R}\left(A+C\right)/I$ (see Appendix A in the extended data ^28^) as a function of α; however, the dynamics approaching equilibrium are different for the two formalisms. Thus, when analyzing short-term dynamics, we assume that only a negligible portion of the population has lost immunity and fix α=1.

**Figure 2.**
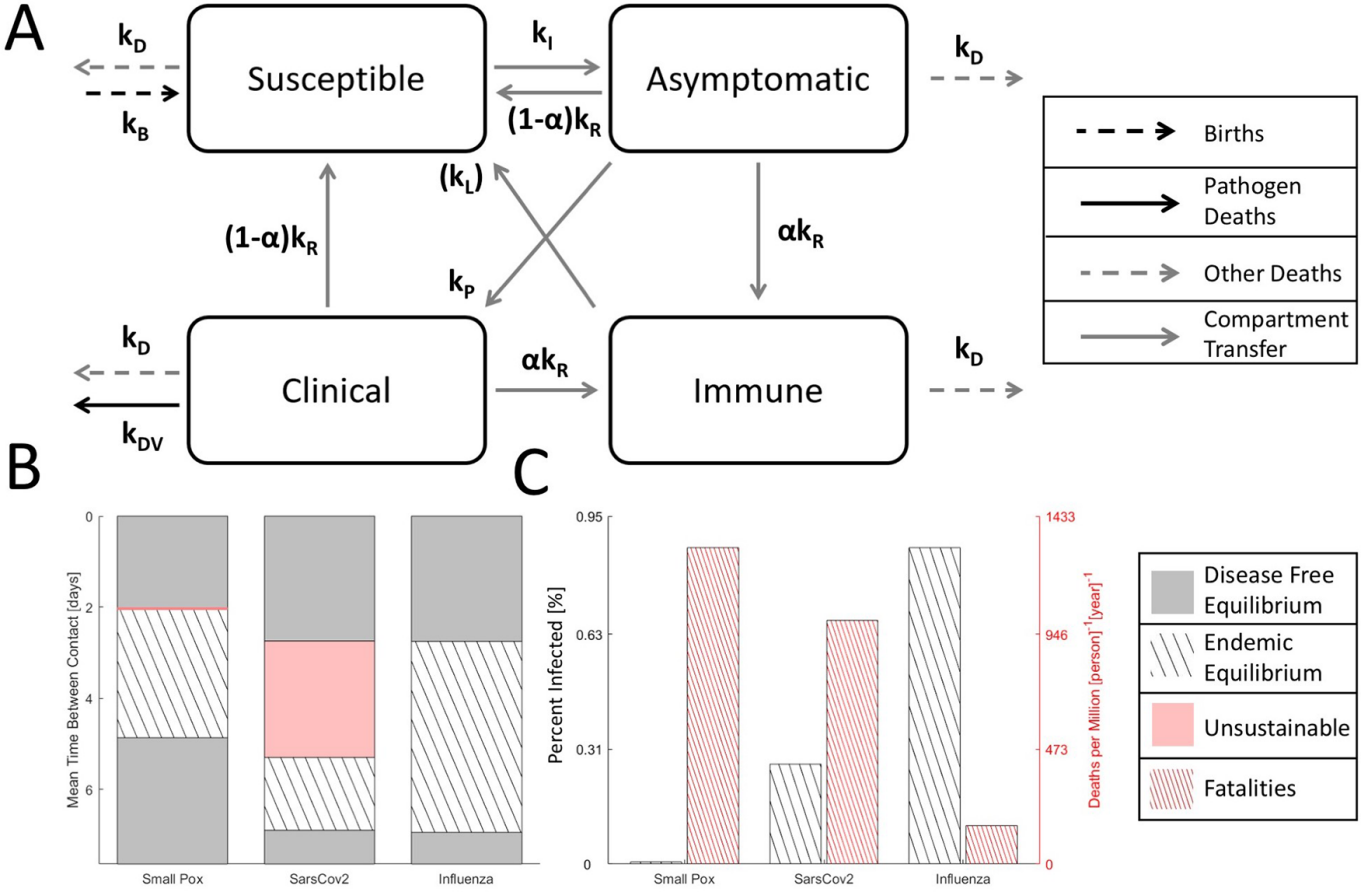
Four compartment model of an epidemic. A. Cartoon of the compartment model. Note that, in the model, permanent immunity is gained with probability *α* for mathematical convenience. Alternatively, temporary immunity can be gained with a probability of 1 and lost at rate *k _L_.* Although the dynamics are different, the corresponding endemic equilibria can be identified by expressing *k _L_* as function of *α* and compartment frequencies. B. Disease free equilibrium (gray), endemic equilibrium (striped), and unsustainable (red) regions for three analyzed viruses over a range of host contact rates. The unsustainable region for smallpox-like viruses is narrow and not shown to scale. C. For endemic equilibrium (contact rates correspond to the midrange shown in B), the fraction of the host population infected and the death rate. Note that, representing equilibrium, the choice of timescale (years) is arbitrary.

The population is assumed to be well mixed, with the exception of the clinical compartment, a fraction $\left(1-\beta \right)$ of which is isolated and cannot infect susceptible hosts. Note this is equivalent to modelling a decreased contact rate for the entire clinical compartment. The rate at which susceptible hosts become infected is the triple product of the rate of contact between hosts, the fraction of the population (not isolated) which is infected, and the probability of infection upon contact: $k_{\text{contact}}\frac{A+\beta C}{I+S+A+\beta C}P\left(\text{infect}\right)$. For simplicity, we consider the product, $k_{I}\equiv k_{\text{contact}}P\left(\text{infect}\right)$, which depends on both host behavior and pathogen biology. Throughout the text, we consider $P\left(\text{infect}\right)=1$ and describe variation in $k_{I}$ as variation in $k_{\text{contact}}$. Lower values of $P\left(\text{infect}\right)$ would not change the bounds on the global phase space or the qualitative trends displayed in any figures; however, they would correspond to higher values of host contact rates than those that appear on the figure axes. This yields the system of ordinary differential equations ( Figure 2A):

$${\left[\begin{array}{c} I\\ S\\ A\\ C \end{array}\right]}^{\prime }=\left[\begin{array}{cccc} -k_{D} & 0 & \alpha k_{R} & \alpha k_{R}\\ k_{B} & k_{B}-k_{D}-k_{I}\frac{A+\beta C}{I+S+A+\beta C} & k_{B}+\left(1-\alpha \right)k_{R} & k_{B}+\left(1-\alpha \right)k_{R}\\ 0 & k_{I}\frac{A+\beta C}{I+S+A+\beta C} & -\left(k_{R}+k_{D}+k_{P}\right) & 0\\ 0 & 0 & k_{P} & -\left(k_{R}+k_{D}+k_{DV}\right) \end{array}\right]\left[\begin{array}{c} I\\ S\\ A\\ C \end{array}\right]$$ (1)

With two infected states (asymptomatic and clinical) and isolation, ISAC is a simple model within the range of models ^29^^–^^31^ that have been developed in response to the SARS epidemic.

Short-term dynamics can be inferred from this system of equations, to establish whether a bottleneck in the size of the susceptible population ( Figure 1B), or the entire population representing a host crisis ( Figure 1A), occurs. For the analysis of dynamics within this period, we assume that only a negligible portion of the host population has gained and subsequently lost immunity, setting α=1. To study this case, we explicitly simulate an epidemic beginning with an initial condition where the size of the infected compartments is five orders of magnitude lower than the value corresponding to the endemic equilibrium, in order to capture realistic dynamics for the emergence of a new pathogen. The basic reproduction number, *R _0_*, for this model can be derived through the construction of next generation matrices ^32^ (see Appendix B in the extended data):

$$R_{0}=\frac{k_{1}}{k_{R}+k_{D}+k_{P}}\left(1+\frac{\beta k_{P}}{k_{R}+k_{D}+k_{DV}}\right)$$ (2)

Short-term dynamics are sometimes determined simply by the *R*_0_, value and when *R*_0_ < 1, the pathogen will go extinct. When *R*_0_ > 1, a wide range of dynamics are possible including short term bottlenecks and long-term endemic equilibrium.

In many cases when *R*_0_ > 1 and a bottleneck is avoided, we are interested in examining the stationary points for the above system of equations. Unique, stable stationary points correspond to endemic equilibrium. While uniqueness and stability have been proven for a very broad class of compartment models, ^33^^,^^34^ more generally, this can be numerically demonstrated for any stationary point as is done throughout this work. At endemic equilibrium, the fraction of the total population, *N*, in each compartment, *X*, is constant: ${\left(\frac{X}{N}\right)}^{\prime }=x^{\prime }-xn^{\prime }=0$ (we denote the fraction of each compartment with lowercase letters). Endemic equilibrium corresponds to a stable stationary point for this system of equations. We represent values corresponding to endemic equilibrium with a “*”, to reflect this. We do not assume a constant size of the total population; however, the results of this simplifying assumption are presented in Appendix C (extended data). More generally, $n^{\prime }=k_{B}-k_{D}-ck_{\mathrm{DV}}$ which yields:

$$0={\left[\begin{array}{c} I/N\\ S/N\\ A/N\\ C/N \end{array}\right]}^{\prime }=\left[\begin{array}{cccc} c^{*}k_{DV}-k_{B} & 0 & \alpha k_{R} & \alpha k_{R}\\ k_{B} & c^{*}k_{DV}-k_{I}\frac{a^{*}+\beta c^{*}}{i^{*}+s^{*}+a^{*}+\beta c^{*}} & k_{B}+\left(1-\alpha \right)k_{R} & k_{B}+\left(1-\alpha \right)k_{R}\\ 0 & k_{I}\frac{a^{*}+\beta c^{*}}{i^{*}+s^{*}+a^{*}+\beta c^{*}} & c^{*}k_{DV}-\left(k_{B}+k_{R}+k_{P}\right) & 0\\ 0 & 0 & k_{P} & c^{*}k_{DV}-\left(k_{B}+k_{R}+k_{DV}\right) \end{array}\right]\left[\begin{array}{c} i^{*}\\ s^{*}\\ a^{*}\\ c^{*} \end{array}\right]$$ (3)

We are primarily interested in establishing the fraction of hosts that are infected $\left(i^{*}+c^{*}\right)\;$ and the number of deaths caused by the pathogen per year $\left(k_{\mathrm{DV}}c^{*}\right)\;$ at endemic equilibrium and, generally, aim to algebraically solve this system of equations for the relative size of each compartment. This system can be solved to yield a fourth order polynomial with respect to c ^*^ (we used the MATLAB symbolic toolbox (RRID:SCR_001622), ^35^ an open-source alternative is Julia, see Appendix D in extended data); however, it is cumbersome enough that a numerical solution appears preferable and establishing opportunities for linearization is desirable.

Endemic equilibrium requires a constant or growing population, limiting the total number of deaths due to infection that can be sustained by the host population per unit time and, accordingly, provides an upper size bound for the clinical compartment. $\frac{k_{B}-k_{D}}{k_{\mathrm{DV}}}\geq c^{*};\frac{k_{B}-k_{D}}{k_{R}}\geq c\frac{k_{\mathrm{DV}}}{k_{R}}$. For human populations and pathogens, it is reasonable to assume that the birth rate is much lower than the recovery rate, $\frac{k_{B},k_{D}}{k_{R}}\ll 1$, yielding the limit: $c^{*}\frac{k_{\mathrm{DV}}}{k_{R}}\ll 1$. Conversely, populations with higher birth rates admit endemic equilibrium for more virulent pathogens, and even among human populations, the host population size plays a critical role in the determination of whether acute pathogens persist. ^18^

Thus, for an endemic equilibrium to exist, either the clinical compartment has to be very small or the death rate due to the virus has to be very low compared to the recovery rate (again, considering the opposite limit, when the birth rate is high, parameter regimes exist where neither endemic equilibrium is reached nor does either the host population or the virus go extinct). This allows us to linearize the model with respect to either the size of the clinical compartment, for pathogens with high CFR, or the ratio of the death rate to the recovery rate, for low CFR pathogens. For the high CFR case, we solve the above system of algebraic equations to first order in $c^{*}$ (e.g. ${\left(c^{*}\right)}^{2}+c^{*}∼c^{*}$, where by definition $0<c^{*}<1$, see Appendix E in the extended data). This linearized model admits an analytic solution for endemic equilibrium, given a sufficiently large *α* corresponding to at least partial immunity whenever $R_{0}>1$. In this case, the additional constraint $k_{P}>k_{R}+k_{\mathrm{DV}}$, which largely holds for the pathogens considered here, is applied for convenience. For the low CFR case, we solve the above system of equations to first order in $\frac{k_{\mathrm{DV}}}{k_{R}}$ (see Appendix F in the extended data). Here the stability of the solution depends on the parameters (it does not belong to the class of models referenced above). Both the stability and the general solution for the critical point are calculated numerically (see Appendix G in the extended data); however, analytic forms for endemic equilibria in the stricter limit $c^{*}\frac{k_{\mathrm{DV}}}{k_{B}}\ll 1$ are provided as well.

Parameters are referenced against those selected to represent three respiratory pathogenic viruses: smallpox, SARS-CoV-2, and influenza representing a range of phenotypes ( Table 2) ^19^^–^^27^ in agreement with the ranges reported for each parameter by the selected references. We emphasize, however, that given the generality of the model, we are unable to make specific predictions for any of these viruses in a defined host population. We instead contrast the results for “smallpox-like” (high CFR), “SARS-CoV-2-like” (intermediate CFR), and “influenza-like” (low CFR) viruses. Here, *k _B_* and *k _D_* are fixed whereas *k _I_* is varied; however, we will emphasize that throughout the text, we consider $P\left(\text{infect}\right)=1$ and describe variation in $k_{I}$ as variation in $k_{\text{contact}}$. *k _R_* and *k _P_* are fit to an estimated disease course for a host which is asymptomatic and infectious for the time $t_{P}=1/k_{P}$, and symptomatic and infectious for the time $t_{R}=1/k_{R}$ before recovering. For simplicity, death due to infection is assumed to occur only during the symptomatic and infectious phase: $k_{\mathrm{DV}}=\frac{\mathrm{CFR}}{1-\mathrm{CFR}}k_{R}$. Smallpox is modelled with a CFR of 30%, mean time to recovery 1 week, and no asymptomatic and infectious period. SARS-CoV-2 is modelled at a CFR of 1%, 1 week recovery period, and 3 days asymptomatic and infectious. Influenza is modelled at a CFR of 0.05%, 1 week recovery period, and 3 days asymptomatic and infectious. Smallpox infection is assumed to confer permanent immunity. SARS-CoV-2 and influenza infections are assumed to confer immunity for one year on average. A constant birthrate of 2.5 births per 2 people over 100 years and death rate of one death per person over 100 years is assumed.

**Table 2.** Human respiratory viruses with substantial health impact.

Name	Case Fatality Rate	Time Asym. N.Inf. (days)	Asym. Inf.	Time Sym. (days)
MERS-CoV	0.35	2–14	minimal	up to 90
Variola major (smallpox)	0.3	7–19	assumed no	9–11
SARS-CoV	0.15	6	assumed no	20
Measles morbilivirus	0.04-0.1	6–21	< one week	6–8
SARS-CoV-2	<0.02	5–14	< one week	21
Influenza	<0.002	2	< one week	3
Mumps rubulavirus	near 0	16–18	< one week	6–9
Rubella virus	near 0	14–18	up to two weeks	largely asymptomatic, symptomatic period largely not infectious
Rhinovirus	near 0	Time course varies, can be infectious for weeks, or wholly asymptomatic, or symptomatic on the first day of infectious period.		
Human respiratory syncytial virus	near 0	Can be wholly asymptomatic		
Human parainfluenza	near 0	Can be wholly asymptomatic		
Alphacoronavirus	near 0	Time course varies, can be wholly asymptomatic		

## Results

Endemic equilibrium, where an unchanging fraction of the host population remains infected in the long term, is bounded within a range of (host) contact rates ( Figure 2B). When the mean time between contacts is too long and *k _I_* is too low, $k_{I}=\left(k_{R}+k_{D}+k_{P}\right){\left(1+\frac{\beta k_{P}}{\left(k_{R}+k_{D}+k_{\mathrm{DV}}\right)}\right)}^{-1}$, *R*_0_ < 1, the pathogen goes extinct, and disease-free equilibrium, where there is no remaining pathogen, is reached. Likewise, when contacts are too frequent, a bottleneck in the susceptible population occurs ( Figure 1B, here assumed to drop down to 10% of the total population), and the pathogen goes extinct. For some viruses, such as smallpox-like and SARS-CoV-2-like viruses, but not influenza-like viruses, a range of contact rates exists where the fraction of the infected population at endemic equilibrium is large enough so that the death rate exceeds the birth rate and the host–virus relationship is unsustainable in the long term, resulting in population decline without decreased pathogen CFR or modified host behavior. This range is very narrow for smallpox-like viruses but notably broad for SARS-CoV-2-like viruses ( Figure 2B, not shown to scale) encompassing a wider range of host behavior than endemic equilibrium. The existence of this region in the parameter space implies that herd immunity, without vaccination, might be impossible to reach, at least, in some segments of the parameter space. In the middle range of contact rates admitting endemic equilibrium ( Figure 1C), the decreased fraction of the infected population for all three examined viruses is offset by increasing CFR leading to an increased death rate. Under these model assumptions, the yearly death rate for a SARS-CoV-2-like virus is approximately 6-times that of an influenza-like virus.

**Figure 3.**
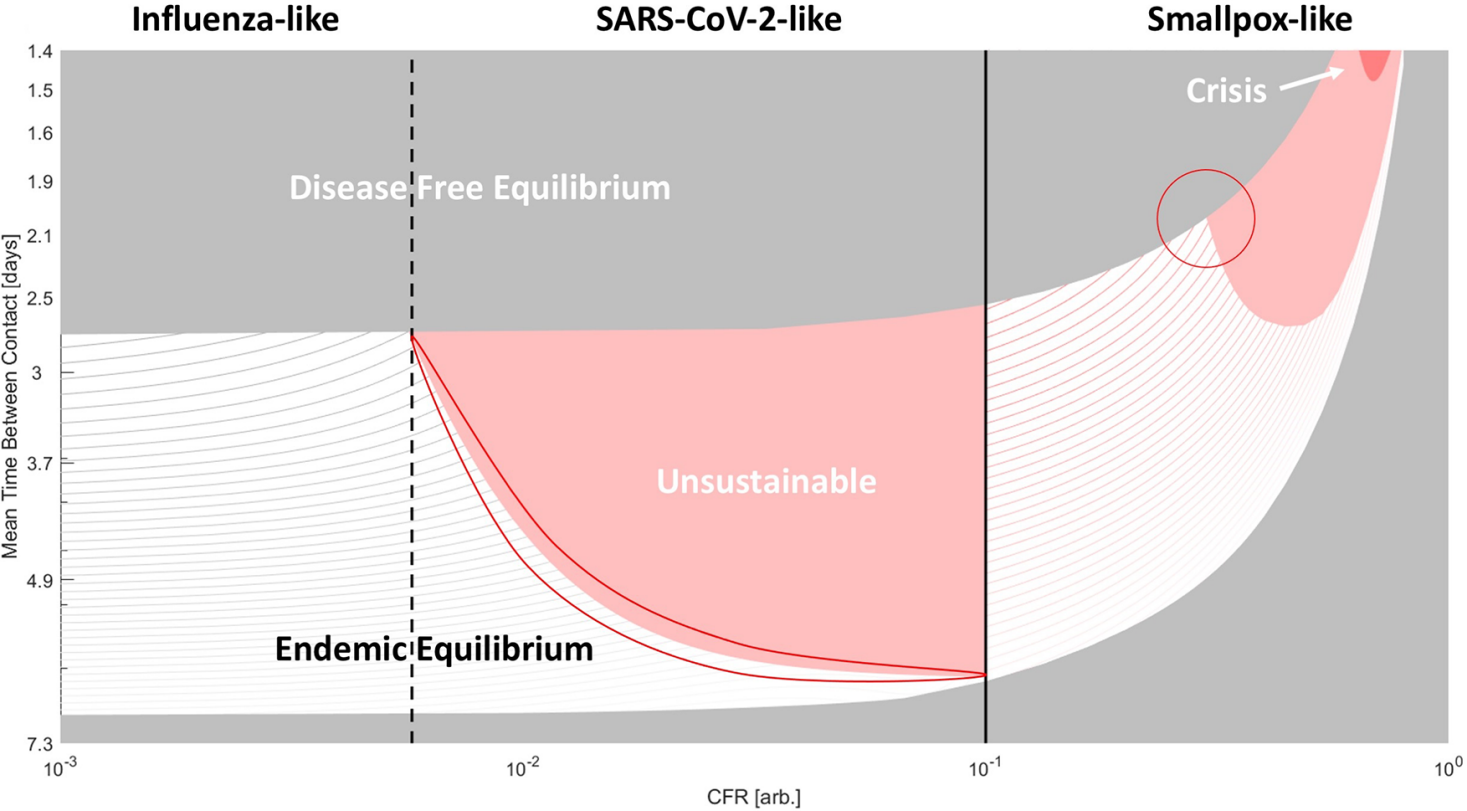
Phase diagram of epidemiological outcomes. The CFR and the contact rate were varied. For variable recovery rate, see Figure S1. Disease-free equilibrium (gray), endemic equilibrium (striped), unsustainable (red), and host population crisis (loss of 90%, dark red) regions are shown. For CFR > 10%, smallpox-like features are assumed. For CFR<10% SARS-CoV-2/influenza-like features are assumed (these viruses are only distinguished within the model by CFR). Lines within the region bounding endemic equilibrium indicate contours for the percentage of the population infected ( a ^*^ + c ^*^)*100. Darker color corresponds to higher values. Two regions subject to kinetic constraints are outlined.

To examine an expanded two-dimensional phase space, we allowed the CFR to vary from 10% to 100% for smallpox-like viruses, with no asymptomatic spread and permanent immunity, and from 0% to 10%, for SARS-CoV-2-like and influenza-like viruses, with asymptomatic spread and temporary immunity ( Figure 3). CFR, host–host contact rate, and the duration of immunity, which is explored in the next figure, are the three most relevant parameters for the study of human pathogenic respiratory viruses apparent from the observed variability among known pathogens ( Table 2). However, it is important to acknowledge the effects of the rate of recovery on the global phase space as well. Throughout the text, we assume a 1-week recovery period. In a supplemental figure (see extended data), we display the results for two additional values (3 days/2 weeks, Figure S1. A/B). The dependency on CFR is remarkably similar across all three recovery regimes, but as one would expect, slower rates of recovery correspond to lower host-host contact rates required to achieve endemic equilibrium.

As the CFR increases, both threshold contact rates, corresponding to *R*_0_ = 1 and to the bottleneck in the susceptible population respectively, increase and the range admitting endemic equilibrium narrows. Across much of the phase space, the host-pathogen relationship is unsustainable, and at very high CFR, the total host population falls below 10% (dark red) in the initial phase of the epidemic, signaling possible extinction at short timescales. The contours within the region corresponding to the endemic equilibrium indicate the total size of the infected host population (which is proportional to the size of the viral population and constitutes our measure of virus fitness). Within this region, increasing contact rate and decreasing CFR increases the size of the infected population. At extremely high CFR, the gradient points primarily in the direction of decreasing CFR, and at low contact rates, the gradient points primarily in the direction of increasing contact rate.

Throughout most of the region in this phase space corresponding to endemic equilibrium for smallpox-like viruses ( Figure 3), moving down and to the left increases the size of the infected population and suggests evolution towards decreased CFR. However, this is not the case at the top-right corner representing viruses with extremely high CFR and extremely high contact rates (or alternatively viruses with a much higher likelihood of infection following host contact). Such, hypothetical, viruses would have an unsustainable relationship with the host population if CFR decreased (and infected hosts were less likely to die before interacting with uninfected hosts) and are, in a sense, kinetically constrained, likely, by the host behavior. The population size of such viruses would dramatically increase if the CFR was reduced but would remain in endemic equilibrium only if contact rates or the rate of infection simultaneously decreased. Smallpox evolution could be similarly kinetically constrained. On the phase diagram for high-CFR viruses, smallpox, with a CFR of 30%, is located near the triple point for host populations with high contact rates where the regions of disease-free equilibrium, endemic equilibrium and unsustainability meet (highlighted in Figure 3). To increase the size of the infected population, both CFR and contact rate must decrease, whereas decreasing only the CFR ultimately results in disease-free equilibrium when such a virus enters new host communities, due to a bottleneck in the size of the susceptible population.

**Figure 4.**
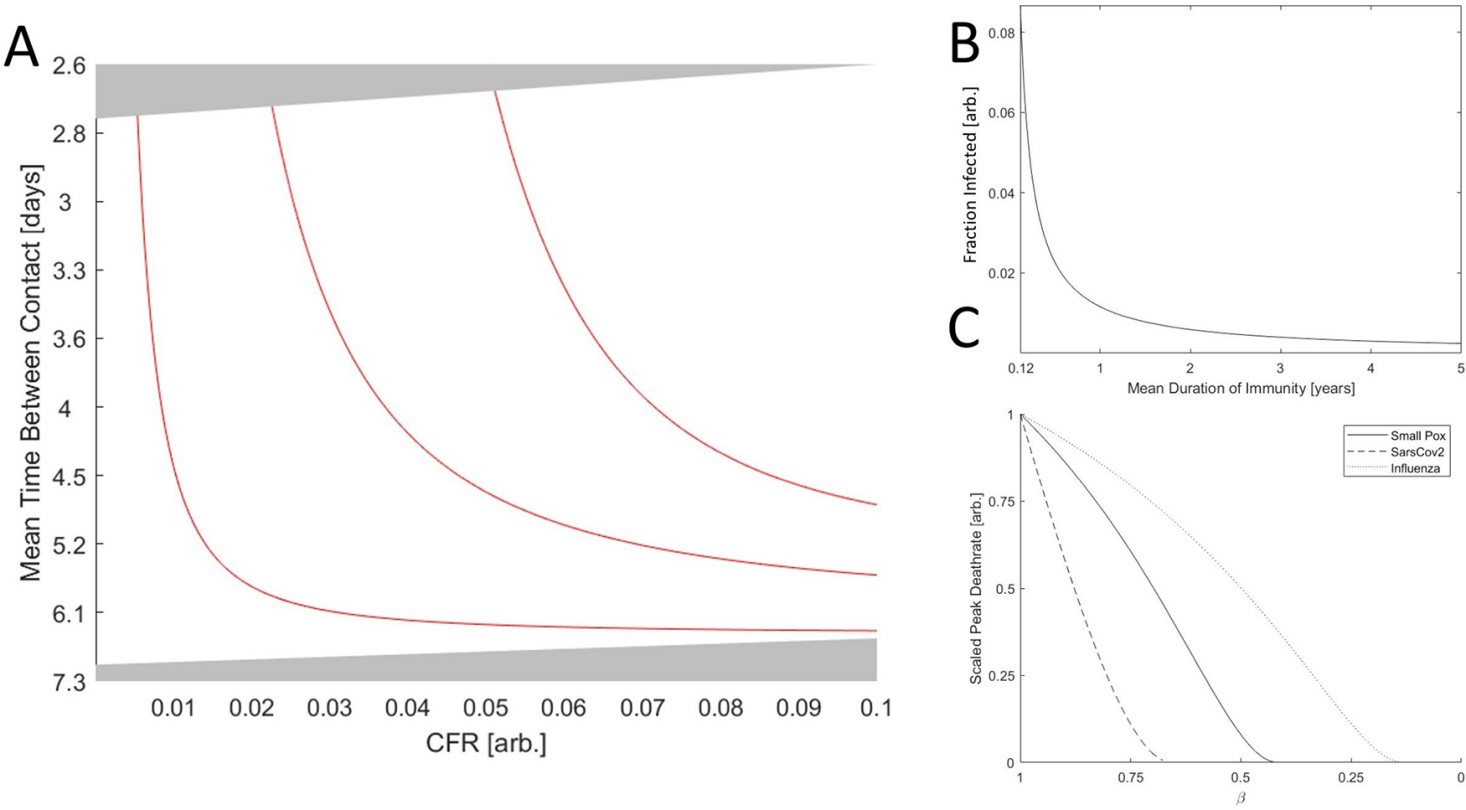
The role of immunity in epidemic evolution. A. Phase diagram of a SARS-CoV-2-like virus varying CFR and contact rate. Red lines indicate the boundary between endemic equilibrium and unsustainable for *α* ∈ {0.01,0.045,0.08} regions corresponding to approximately {1.5,7,13} years of immunity, respectively. B. The fraction of the population infected, ( a ^*^ + c ^*^), vs the duration of immunity for a hypothetical virus with a CFR of zero (other parameters matching influenza and Sars-Cov-2) and the maximum admitted contact rate for endemic equilibrium. C. Peak death rate at the height of an epidemic with contact rate corresponding to the maximum value admitting endemic equilibrium given *β* = 1 and varying *β* (decreasing *β* corresponds to increasing isolation). Death rate is scaled by the maximum value for each virus.

The corresponding triple point located at the boundary between influenza-like and SARS-CoV-2-like viruses lacks this property. For these viruses, decreasing CFR increases the size of the infected population throughout the parameter space. However, the boundary highlighted within the SARS-CoV-2-like diagram ( Figure 3) represents a different kinetic constraint, this one, between immunity and CFR. While decreasing CFR below 10% minimally affects the threshold contact rates ( *R*_0_ = 1 and susceptibility bottleneck), varying the duration of immunity has a dramatic impact on the phase diagram ( Figure 4A). As the duration of immunity decreases, with α=0.01 corresponding to approximately 1.5 years at the phase boundary, the endemic equilibrium region shrinks substantially.

As is apparent from the analytic solutions given in the extended data and illustrated for a hypothetical virus with a CFR of zero (other parameters matching influenza and Sars-Cov-2) and the maximum admitted contact rate ( Figure 4B), decreasing the duration of immunity dramatically increases the size of the infected population at endemic equilibrium across the parameter regimes. While typically viewed from a host-centric perspective, immunity is almost always necessary for the maintenance of endemic equilibrium and thus required for maintaining large viral populations over long time scales. Consider a SARS-CoV-2-like virus near the highlighted boundary in Figure 3. Suppose this virus acquires an adaptation enabling immune evasion and thus decreasing the mean duration of immunity post infection. The size of the infected population will increase and, being near the boundary, the host–pathogen relationship will become unsustainable. This is another example of a kinetic constraint. Decreasing both CFR and the duration of immunity increases the size of the infected host population. However, maintaining a stable host–pathogen relationship and large numbers of infected hosts in the long term requires that the reduction in CFR is proportional to the reduction in immune duration. Otherwise, the overall death rate for the host population might increase despite a reduction in pathogen CFR. Notably, decreasing the host–host contact rate moves the population farther from this boundary in the phase space and alleviates this kinetic constraint. Therefore, even if host–host contact rates cannot be reduced enough to break endemic equilibrium by reaching *R*_0_ < 1, it’s possible that a modest reduction can change the evolutionary trajectory of the pathogen from one of stagnant or increasing CFR to one of decreasing CFR.

**Figure 5.**
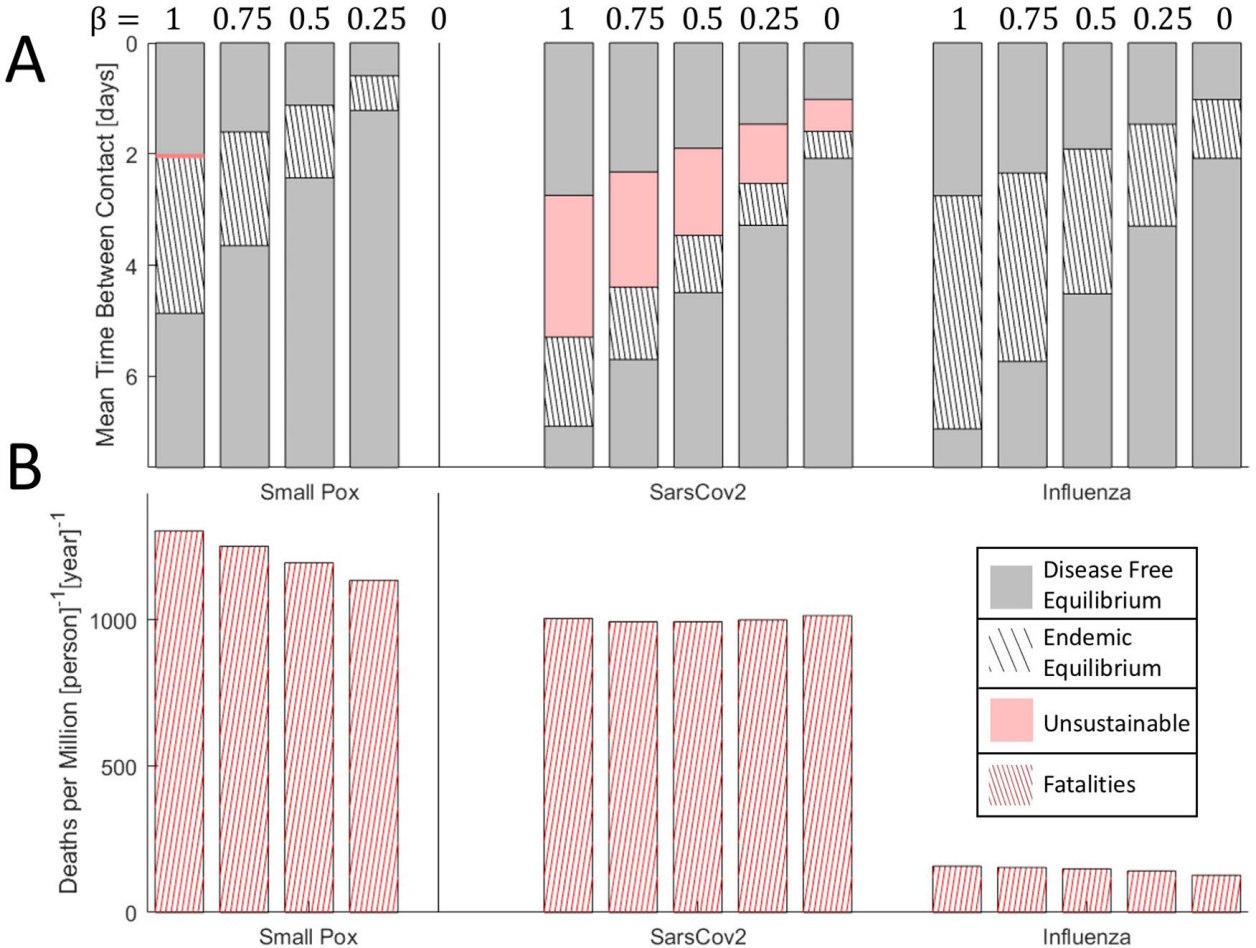
The effects of isolation on epidemic evolution. A. Disease free equilibrium (gray), endemic equilibrium (striped), and unsustainable (red) regions for three viruses over a range of host contact rates. Red region for smallpox-like viruses is narrow and not shown to scale. Bars are ordered by decreasing *β* ∈ {0,0.25,0.5,0.75,1}. B. Death rate at endemic equilibrium (contact rates correspond to the midrange shown in A). Bars are ordered by decreasing *β* ∈ {0,0.25,0.5,0.75,1}. Note that, representing equilibrium, the choice of timescale (years) is arbitrary.

One way a host population can effectively decrease the rate of infection is through the isolation of symptomatic individuals. Isolation decreases death rate at the peak of the epidemic ( Figure 4C) and can prevent an epidemic entirely by driving *R*_0_ below 1, which requires $\beta <\left(\frac{k_{R}+k_{D}+k_{P}}{k_{I}}-1\right)\left(\frac{k_{R}+k_{D}+k_{\mathrm{DV}}}{k_{P}}\right)$. For pathogens with substantial asymptomatic or pre-symptomatic spread (small $k_{P}$), this approach might not be feasible. Although isolation narrows the range of contact rates that admits endemic equilibria or unsustainability ( Figure 5A), the death rate at endemic equilibrium varies little with decreasing *β* and may even increase in the case of SARS-CoV-2-like viruses. Notably, however, SARS-CoV-2-like viruses are particularly sensitive to changes in *β* such that a modest decrease in *β*, that is, increased isolation, can change the long-term outcome from endemic equilibrium to disease-free equilibrium. Furthermore, despite a 30-fold difference in CFR, both smallpox-like viruses and SARS-CoV-2-like viruses lead to the death of approximately 0.1% of the host population per year at endemic equilibrium. This highlights how pathogens with low or intermediate CFR can cause as many fatalities as a pathogen with a much higher CFR if allowed to reach endemic equilibrium.

## Discussion

We show here that CFR evolution in human pathogenic respiratory viruses can often be kinetically constrained. However, these constraints represent only the most general limitations on potential evolutionary trajectories. We did not conduct an invasion analysis, and many paths that apparently are not constrained in the global phase space ( Figure 3) are inaccessible under specific conditions. The accessibility of these trajectories depends on both the global bounds to endemic equilibrium discussed here and the gradient of pathogen fitness. We consider the total number of infected hosts as our measure of pathogen fitness, which is only viable for establishing long-term trends. Different measures of fitness are required to determine barriers to invasion. ^36^

Nonetheless, even these most general constraints likely play key roles in shaping the host–pathogen relationship of important human respiratory viruses. A reduction in CFR increases the average amount of time an infected host spends in the clinical compartment. This increases the total number of new infections that result from contact with each infected host. For smallpox-like pathogens with high CFR in communities with frequent contact, this can create a bottleneck in the size of the susceptible population, resulting in disease-free equilibrium (that is, extinction of the virus) when not accompanied by a decrease in the rate of infection. The rate of infection is determined by both the probability of infection given host–host contact and the host–host contact rate. Under these conditions, if the probability of infection following host contact were internally constrained by CFR, this would not constitute a fitness trade-off and could facilitate host adaptation. On the other hand, high host–host contact rates could externally constrain the rate of infection making reduction in CFR costly to the virus. Evolution of the smallpox virus shows a steady pattern of gene losses that likely lead to increasing infectivity and virulence. ^37^^,^^38^ This evolutionary trend appears to be compatible with the conclusion that high CFR, smallpox-like viruses are unlikely to evolve towards decreasing CFR due to constraints imposed by the host behavior. Similar constraints have likely led to the emergence of acuteness for human bacterial respiratory pathogens of the genus *Bordetella*. ^18^

For SARS-CoV-2-like pathogens with moderate CFR, evolution towards decreased CFR can be kinetically constrained by the relationship between CFR and the duration of immunity. Decreasing the duration of immunity increases the size of the infected population and the overall death rate which can make the host–pathogen relationship unsustainable. The existence of a large region of the phase space corresponding to unsustainable or kinetically constrained moderate-CFR viruses implies two distinct forms of host response over two different timescales. Over long timescales, unsustainable viruses are likely to face extinction due to the elimination of the susceptible host population. Although, in principle, this could occur *via* extinction of the entire host population, the emergence of host resistance is likely. Over short timescales, especially for modern human populations, the emergence of an unsustainable virus, such as SARS-CoV-2, may be considered societally unacceptable, leading to drastic measures that result in a major reduction in host–host contact rates. Both trends likely contribute to the paucity of moderate CFR human respiratory viruses which are subject to this kinetic constraint between immunity and CFR.

Perhaps paradoxically, immune evasion could incur a fitness cost for the pathogen and even lead to its extinction due to the host response. However, some level of immune evasion is required, among host populations with a low birthrate, to maintain any state of endemic equilibrium in the case where lifelong immunity is conferred against individual strains and the duration of immunity is determined by antigenic drift rather than the decline of immunity itself. Evolution towards decreased CFR is uncertain in this case, and a better understanding of internal genomic constraints ^7^ could help predict the effects of immunomodulation. This is important when assessing the impact of novel or imperfect vaccination which can lead to counterintuitive results. ^15^^,^^39^^,^^40^ Diversification related to immune evasion commonly enables the maintenance of large virus populations over long time scales, as is the case for influenza. ^41^^–^^43^ In the case of SARS-CoV-2, immune evasion, diversification, and host adaptation of SARS-CoV-2 have already been demonstrated. ^44^^–^^46^ Furthermore, products of virus genes that are specifically found in pathogenic beta-coronaviruses have been implicated in immunomodulation, ^47^ suggesting that the virus adapts to maintain an endemic equilibrium in this way. Although the present model cannot predict whether SARS-CoV-2 will become more or less virulent, our results do suggest that evolution of its CFR is kinetically constrained such that the region of the parameter space where reduced CFR could evolve is small. On the other hand, we show that even modestly decreasing the host–host contact rate can alleviate this kinetic constraint and promote CFR reduction.

During both the ongoing SARS-CoV-2 pandemic and the first SARS-CoV-1 epidemic, stringent public health measures were taken to limit infection, extending beyond isolation of symptomatic individuals and into the quarantine of asymptomatic, and likely uninfected, contacts. ^22^^,^^31^ Although only a crude depiction of the nuanced dynamics underlying SARS-CoV-2 infections, the analysis presented here suggests that isolation and quarantine are particularly effective towards changing the long-term outcome for viruses with moderate CFR and high probability of infection following host contact, such as SARS-CoV-2. The phase diagram for SARS-CoV-2-like viruses is sensitive to the parameter *β* which reflects the isolation of symptomatic individuals (and additionally asymptomatic carriers who have tested positive). The evolution of the epidemic for such viruses is dominated by disease-free equilibrium or an unsustainable host–virus relationship. Endemic equilibrium is possible only in a narrow parameter range and is therefore unlikely. Nonetheless, the existence of this range suggests that herd immunity is unlikely in the absence of vaccination and would amount to an extreme number of fatalities. Our analysis shows that, while highly amenable to public health intervention, SARS-CoV-2-like viruses can be expected to contribute to a substantially higher death toll than influenza-like viruses, comparable instead to that of a smallpox-like virus, over a protracted period.

We should emphasize one last time that our aim in this study was to use a maximally general model to chart the global phase space of human respiratory viruses. We did not use any explicit trade-off functions or specify within-host pathogen dynamics. Given the generality and simplicity of this approach, these results cannot be immediately leveraged to predict the outcome of specific epidemics for specific host populations. Such prediction requires an invasion analysis, taking into account many additional factors. ^5^^,^^17^^,^^18^ Furthermore, throughout this work, we discuss ways in which host–pathogen interactions affect evolutionary outcomes. It is also important to acknowledge that environmental influences, perhaps most prominently the stability of the exposed pathogen, ^48^ can additionally constrain the evolutionary outcome of an epidemic. Additionally, the model presented here is deterministic and assumes a large, well-mixed host population. Finite size effects for small or spatially segregated host populations can additionally affect epidemic outcome, ^2^ resulting in substantial stochasticity.

## Conclusions

Human respiratory epidemics often evolve under kinetic constraints. These constraints can prevent the reduction in CFR for both high- and moderate-CFR viruses. The incorporation of these constraints can assist in the interpretation of classical model results for epidemics where some parameters, such as host–host contact rate, are unknown. We show that SARS-CoV-2-like viruses are unlikely to reach a state of endemic equilibrium; however, the potential for such equilibrium implies that herd immunity without vaccination may be impossible. At equilibrium, moderate-CFR viruses can cause as many fatalities as high-CFR viruses with both SARS-CoV-2-like viruses and smallpox-like viruses leading to death of about 0.1% of the population per year. However, even partial isolation of symptomatic individuals can have a major effect not only by reducing the number of fatalities in the short term but also by potentially changing the evolutionary trajectory of the virus towards reduced CFR. Such simple public health interventions can continue to dramatically decrease the forecasted cost of SARS-CoV-2 over both the short and long term.

## Author contributions

Conceptualization and Formal Analysis: NDR, YIW, and EVK

Writing – Original Draft Preparation: NDR and EVK

Writing – Review & Editing: NDR, YIW, and EVK

## Data Availability

Zenodo: Extended Data for ‘Evolution of human respiratory virus epidemics’ https://doi.org/10.5281/zenodo.4818157. ^28^ The project contains the following underlying data:
• Appendix.pdf • FigureS1.pdf Data are available under the terms of the Creative Commons Attribution 4.0 International license (CC-BY 4.0).
