# Supplementary material for: Evolution of human respiratory virus epidemics
Source: F1000Res. 2021 Jul 29;10:447. Originally published 2021 Jun 4. [Version 2] doi: 10.12688/f1000research.53392.2 (PMC8381341; doi:10.12688/f1000research.53392.2)
Supplement: Peer review history

# Peer review: Reviewer response for version 2

**DOI:** 10.5256/f1000research.58799.r90715

**Published:** 2021-08-20

**Authors:** Kim Sneppen

**Affiliations:** 1 Niels Bohr Institute, University of Copenhagen, Copenhagen, Denmark

I am happy with the clarifications provided by the authors.

 As minor comments, I still found the picture of a rate being proportional to activation energy E strange, since rates are rather proportional to exp(-E/Temperature). Also the following paragraph implying an detailed balance for diseases, somewhat misrepresenting SIRS, i.e. a cyclic set of reactions where there is no detailed balance (but of course a steady endemic state). The authors probably work on a more philosophical level here, but that does not make the analogy clearer.

Is the work clearly and accurately presented and does it cite the current literature?

Partly

If applicable, is the statistical analysis and its interpretation appropriate?

Not applicable

Are all the source data underlying the results available to ensure full reproducibility?

Partly

Is the study design appropriate and is the work technically sound?

Partly

Are the conclusions drawn adequately supported by the results?

No

Are sufficient details of methods and analysis provided to allow replication by others?

Partly

Reviewer Expertise:

NA

I confirm that I have read this submission and believe that I have an appropriate level of expertise to confirm that it is of an acceptable scientific standard.

# Peer review: Reviewer response for version 2

**DOI:** 10.5256/f1000research.58799.r90716

**Published:** 2021-08-10

**Authors:** Claus O. Wilke

**Affiliations:** 1 Department of Integrative Biology, The University of Texas, Austin, TX, USA

The article is very much improved after revision and I have no further comments or concerns.

Is the work clearly and accurately presented and does it cite the current literature?

Yes

If applicable, is the statistical analysis and its interpretation appropriate?

Yes

Are all the source data underlying the results available to ensure full reproducibility?

No source data required

Is the study design appropriate and is the work technically sound?

Yes

Are the conclusions drawn adequately supported by the results?

Yes

Are sufficient details of methods and analysis provided to allow replication by others?

Yes

Reviewer Expertise:

mathematical modeling, evolutionary biology, infectious disease

I confirm that I have read this submission and believe that I have an appropriate level of expertise to confirm that it is of an acceptable scientific standard.

# Peer review: Reviewer response for version 1

**DOI:** 10.5256/f1000research.56772.r86917

**Published:** 2021-07-07

**Authors:** Kim Sneppen

**Affiliations:** 1 Niels Bohr Institute, University of Copenhagen, Copenhagen, Denmark

The paper investigates the sustainability of steady state (endemic states) of various diseases. Given the paper is a rather simple modification of a SIRS model, the paper is a remarkable difficult read. Thus I may easily be missing something due to my difficulty in following the logic.

1. The title mention evolution, but I failed to find what is evolving, probably because I could not grasp the true meaning of the kinematic constraints, i.e. what mechanism among the virus should want to drive it towards a steady state endemic state, given that the virus tend to be selected to maximize its offspring from generation to generation? A more clear explanation of this potentially important new scale of evolutionary selection would be helpful.
2. Overall, I am uncertain about the steady state approach to the diseases, as endemic like diseases often comes in pulses, like smallpox in the late 18th century, or perhaps like standard influenza epidemics (with all their caveats on mutations and partial cross-immunity).
3. In more detail, I am sorry, but do not get the logic of Fig. 1. Clearly, a disease can eventually go extinct because either the transmission rate is to low, or so high that one fast reach a state where herd immunity makes further propagation impossible. In between these extremes there will be some endemic state, supported by susceptible from birth or from loss of immunity from previously infected people.
4. Table 2 does not give a hint about real differences in infection rates between smallpox and coronavirus. Apart from that then the parameters from SARS-CoV-2 is misleading, as serial interval and infectious period each is about 5 days.
5. Fig. 2 nicely outlines the model, but then in panel B,C) presents data using a y-axis that quantifies kI(?) in contact days? What about infectivity, some diseases like smallpox have a R0 of about 20, while other like SARS have R0 between 2 and 5. How does the y-axis in Fig. 2 relate to these very different scenarios?
6. Fig. 3 is again a hard figure to read? How can smallpox softly transit from SARS-Cov-2. I do not get how increased case fatality rate changes the regime of the sustainable endemic state? Also, the disease free equilibrium at short contact times presumably reflects situations where the first epidemic was so brutal that herd immunity was more than obtained? I don't understand the unsustainable red region? Why larger for larger CFR?
7. Fig. 4 is difficult given the arbitrariness of all parameters. What is the point of 3 sub-figures, what could I as a reader learn?
8. Fig. 5 investigates mitigation, something that has little to do with endemic states (presumably we would not like to have a permanently mitigated society?). I also failed to locate whether the mitigation fraction beta only included reduction in the symptomatic transmission? For SARS-CoV-2 there is a lot of asymptomatic transmission, but not for SARS.
9. In conclusion, the authors say that SARS-CoV-2 cannot reach a state of endemic equilibrium. They might be right, but one might as well imagine that disease immunity decline sufficiently fast that it could be constantly circulating in some mild form. Also, I cannot see how its increasingly many mutant variants would make such a conclusion robust.
10. In the results section of the abstract the authors state: "We reveal kinetic constraints where the variation of multiple parameters in concert leads to decreased CFR and increased pathogen fitness, whereas independent variation of the parameters decreases pathogen fitness. Smallpox, SARS-CoV-2, and influenza are analyzed as diverse representatives of human respiratory viruses. We show that highly virulent viruses, such as smallpox, are likely often constrained by host behavior, whereas moderately virulent viruses, such as SARS-CoV-2, appear to be typically constrained by the relationship between the duration of immunity and CFR." This is beyond my understanding: smallpox could not really be constrained (or could not when it recurrently broke out in cities in the 18th century). SARS-CoV-2 was in contrast contained by human mitigation, and had little directly to do with CFR.
11. A minor point is the strange discussion of reversible chemical reactions in a paragraph on page 2. It seems to assume that energy is directly proportional to transmission rates. Also its relation to the rest of the manuscript is unclear.
12. Overall, the paper presents a worthwhile investigation into an important phenomenon. However, it is very difficult to read and evaluate given its unclear assumptions (and complicated sentences). I would encourage a major rewrite, emphasizing which few (hopefully) parameters that are important, and  with new figures that more clearly illustrate the main point.

Is the work clearly and accurately presented and does it cite the current literature?

Partly

If applicable, is the statistical analysis and its interpretation appropriate?

Not applicable

Are all the source data underlying the results available to ensure full reproducibility?

Partly

Is the study design appropriate and is the work technically sound?

Partly

Are the conclusions drawn adequately supported by the results?

No

Are sufficient details of methods and analysis provided to allow replication by others?

Partly

Reviewer Expertise:

NA

I confirm that I have read this submission and believe that I have an appropriate level of expertise to state that I do not consider it to be of an acceptable scientific standard, for reasons outlined above.

# Response

**Published:** 2021-07-17

**Authors:** Eugene Koonin

**Affiliations:** National Center for Biotechnology Information (NCBI), National Institutes of Health (NIH), USA

**Response to Kim Sneppen:**

- *The paper investigates the sustainability of steady state (endemic states) of various diseases. Given the paper is a rather simple modification of a SIRS model, the paper is a remarkable difficult read. Thus I may easily be missing something due to my difficulty in following the logic.*
- **Response: **We apologize for any confusion and hope that our revised presentation is substantially clearer.

- *The title mention evolution, but I failed to find what is evolving, probably because I could not grasp the true meaning of the kinematic constraints, i.e. what mechanism among the virus should want to drive it towards a steady state endemic state, given that the virus tend to be selected to maximize its offspring from generation to generation? A more clear explanation of this potentially important new scale of evolutionary selection would be helpful. Overall, I am uncertain about the steady state approach to the diseases, as endemic like diseases often comes in pulses, like smallpox in the late 18th century, or perhaps like standard influenza epidemics (with all their caveats on mutations and partial cross-immunity).*
- **Response: **This study is primarily concerned with very long-term evolutionary outcomes. The reviewer makes the important point, which has been emphasized in the revised version that, in reality, a pathogen rarely reaches a true steady state. Outbreaks are almost always spatially and/or seasonally dependent, and the fraction of a host population that is infected fluctuates significantly over time. However, even diseases like Influenza or Smallpox that are or were subject to periodic epidemics or pandemics, during which the number of infected individuals is much higher than average, both are or were endemic to the human population such that a predictable number of cases would be sustained on average within a year or a decade. This study largely focuses on such averages as emphasized in the revision.

- *In more detail, I am sorry, but do not get the logic of Fig. 1. Clearly, a disease can eventually go extinct because either the transmission rate is to low, or so high that one fast reach a state where herd immunity makes further propagation impossible. In between these extremes there will be some endemic state, supported by susceptible from birth or from loss of immunity from previously infected people.*
- **Response:** Figure 1 was introduced to provide context for a broad audience. The reviewer is correct that the content of this figure may be obvious for an expert reader. Indeed, the logic is described correctly in the reviewer’s comment. We still think that this figure is useful for a reader unfamiliar with specifics of evolutionary epidemiology, and under the current circumstances, we expect there will be many such readers of this paper.

- *Table 2 does not give a hint about real differences in infection rates between smallpox and coronavirus. Apart from that then the parameters from SARS-CoV-2 is misleading, as serial interval and infectious period each is about 5 days.*
- **Response: **The reviewer is correct that Table 2 does not list the differences in the rates of infection. This is because the rate of infection is the triple product of the rate of contact between hosts, the fraction of the population (not isolated) which is infected, and the probability of infection upon contact. Notwithstanding the variability in the probability of infection upon contact, which itself depends on the viral load of the infected individual and the nature of contact, substantial variability of the rate of contact between hosts makes it extremely challenging to report globally meaningful rates of infection for pathogens, and especially, respiratory pathogens. We agree that this table could be misleading considering the large values reported for the time symptomatic, which does not reflect the fact that hosts may be symptomatic but no longer infectious. This has been clarified in the text. SARS-CoV-2 is modelled with a 7 day period, during which the host is infectious and symptomatic, preceded by a 3 day asymptomatic and infectious period. This was the consensus prediction at the time this manuscript was initially prepared and we have acknowledged in the revised text that, given its recent identification, the best fit parameter values for SARS-CoV-2 are subject to change. We do not model the serial interval because, like the rate of infection, it depends on the rate of contact.

- *Fig. 2 nicely outlines the model, but then in panel B,C) presents data using a y-axis that quantifies kI(?) in contact days? What about infectivity, some diseases like smallpox have a R0 of about 20, while other like SARS have R0 between 2 and 5. How does the y-axis in Fig. 2 relate to these very different scenarios?*
- **Response: **We thank the reviewer for their appreciation of this figure and regret any possible confusion. As discussed in the previous comment, the rate of infection is the triple product of the rate of contact between hosts, the fraction of the population (not isolated) which is infected, and the probability of infection upon contact. kI, as listed in Table 1, is the product of the rate of contact and the probability of infection upon contact: kcontact*P (infect). In principle, both may vary, so for simplicity, we study this compound parameter, which can increase as a result of either increasing P(infect) or increasing kcontact. As P(infect) is unitless, kI has units of 1/time and varying kI is equivalent to varying the time between contact, which is what we report. While this was stated in the original version where the model is initially described, we acknowledge the presentation could use improvement and apologize for any confusion. We have added additional clarification on the nature of kI and the units of the y-axis in the figures. Additionally, we note that R0 depends on the rate of host contact and so will also vary across communities and over time for a single pathogen.

- *Fig. 3 is again a hard figure to read? How can smallpox softly transit from SARS-Cov-2. I do not get how increased case fatality rate changes the regime of the sustainable endemic state? Also, the disease free equilibrium at short contact times presumably reflects situations where the first epidemic was so brutal that herd immunity was more than obtained? I don't understand the unsustainable red region? Why larger for larger CFR?*
- **Response: **We regret the confusion. The reviewer is completely correct that smallpox cannot smoothly transit from SARS-CoV-2. This is evident in Figure 3 because there is no path from the smallpox-like regime to the SARS-CoV-2-like regime that does not exit endemic equilibrium. The solid line in the plot indicates that to the right of the line, the pathogen confers permanent immunity and to the left of the line, temporary immunity. The dashed line represents a smooth transition between the SARS-CoV-2-like regime and the Influenza-like regime, which are both modelled to produce temporary immunity. This has been clarified in the revised version. The duration of immunity is the third key parameter that is varied in Figure 4A. Increased CFR changes the regime of the sustainable endemic state because it changes the balance between births and deaths in the population. An endemic pathogen with a high CFR results in a greater number of mortalities per year for the host population. The reviewer is correct that disease-free equilibrium at short contact times reflects a selective bottleneck in the host population as illustrated in Figure 1B. This has been emphasized in the revised version. However, it should be noted that a selective bottleneck at low CFR does not necessarily represent an outbreak resulting in significant mortality. The unsustainable red region represents the case where endemic equilibrium is possible but eventually, because the number of deaths sustained by the pathogen exceeds the birth rate, the host population will either go extinct or must adapt in some way to avoid the pathogen. This represents an unsustainable host-pathogen relationship. This region is larger for larger CFR because the number of deaths sustained by the population increases with increasing CFR.

- *Fig. 4 is difficult given the arbitrariness of all parameters. What is the point of 3 sub-figures, what could I as a reader learn?*
- **Response: **We respectfully suggest that the parameters are not arbitrary. Figures 3 and 4A are critical to understanding the life history phase space that is available human respiratory viruses. Our choice of parameter exploration in the initial submission was motivated by the range of parameters observed for pathogenic human respiratory viruses. Additionally, where possible, analytic dependencies on the size of the clinical population at endemic equilibrium are presented in the Appendix. These pathogens span a wide range of CFR, and host-host contact rates can vary significantly among human populations. We additionally vary the amount of time a host is both asymptomatic and infectious although we do not allow this parameter to vary freely because that would have been implausible from a biological standpoint: hosts infected with high CFR viruses are not known to spend significant time in an asymptomatic and infectious state. We additionally explore the full range of immune duration (parametrized by 0<=alpha<=1) for SARS-CoV-2-like and Influenza-like viruses. This parameter largely determines the region of the phase space that corresponds to unsustainability, and representative values are shown in red curves in figure 4A. This covers the CFR (k_DV), the rate of progression (k_P), the rate of contact/probability of infection (k_I), and the rate at which immunity is lost (k_L). We discuss the importance of small birth rate, and a small difference between k_B and k_D (deathrate not specific to the pathogen), for interpreting our results and linearization of the model requires that value to be small. These rates are expected to vary significantly less among human populations than pathogen-specific rates and we submit are not critical to vary. Lastly, we devote the final figure to studying the effects of the parameter beta representing isolation of symptomatic individuals. Thus we hope we have demonstrated to the reviewer that we effectively surveyed the entire parameter space admitting endemic equilibrium for the pathogens of interest. Additionally, although we continue to maintain that the parameters fully explored in the main text constitute those of greatest relevance given the known variability among human respiratory viruses, we acknowledge that a demonstration of the dependence on k_R is useful for the reader as well. This is available as a supplementary figure. We agree that panels B and C are perhaps less critical to understanding the key results of this study, but still contain information central to what is discussed in the text. Specifically, panel B demonstrates that the size of the viral population at endemic equilibrium decreases with an increasing duration of immunity. Panel C demonstrates the reduced cost of an epidemic resulting in reducing the contact rate among symptomatic hosts and is a segue into the final figure.

- *Fig. 5 investigates mitigation, something that has little to do with endemic states (presumably we would not like to have a permanently mitigated society?). I also failed to locate whether the mitigation fraction beta only included reduction in the symptomatic transmission? For SARS-CoV-2 there is a lot of asymptomatic transmission, but not for SARS.*
- **Response: **We regret any confusion. Mitigation may refer to either the isolation of symptomatic individuals or the quarantine of asymptomatic, and likely uninfected, contacts. While we discussed this in the original version, this has been emphasized in the revised version. We only model the former – isolation of symptomatic hosts and would argue that in that sense we already live in a “permanently mitigated society”. Such mitigation has a dramatic effect on the range of host-host contact rates which admit endemic equilibrium as displayed in Figure 5A. The reviewer is correct that the death rate at endemic equilibrium is insensitive to mitigation. This is displayed in Figure 5B. The reviewer is also correct that asymptomatic transmission for SARS-CoV-2 is more substantial than it was for SARS. We discuss how high CFR pathogens are generally subject to less asymptomatic transmission than those with lower CFR.

- *In conclusion, the authors say that SARS-CoV-2 cannot reach a state of endemic equilibrium. They might be right, but one might as well imagine that disease immunity decline sufficiently fast that it could be constantly circulating in some mild form. Also, I cannot see how its increasingly many mutant variants would make such a conclusion robust.*
- **Response:** We agree that the wording in the abstract was somewhat overstated, so we have amended it in the revision. In the main text we state, “We show that SARS-CoV-2-like viruses are unlikely to reach a state of endemic equilibrium; however, the potential for such equilibrium implies that herd immunity without vaccination might not be achievable”. We do not intend to state SARS-CoV-2 “cannot” reach a state of endemic equilibrium. The reviewer suggests that the duration of immunity may decline sufficiently fast so that the pathogen could circulate in some mild form. This implicitly assumes that, in addition to a reduction in the duration of immunity, SARS-CoV-2 would also evolve towards a reduction in CFR. This is precisely what we discuss. Regarding the emergence of novel variants, we think it is important to emphasize that antigenic drift is a major driver of endemic equilibrium, and furthermore, that all SARS-CoV-2 variants still firmly reside in the SARS-CoV-2-like region of the global phase space. We have clarified this point in the revised text.

- *In the results section of the abstract the authors state: "We reveal kinetic constraints where the variation of multiple parameters in concert leads to decreased CFR and increased pathogen fitness, whereas independent variation of the parameters decreases pathogen fitness. Smallpox, SARS-CoV-2, and influenza are analyzed as diverse representatives of human respiratory viruses. We show that highly virulent viruses, such as smallpox, are likely often constrained by host behavior, whereas moderately virulent viruses, such as SARS-CoV-2, appear to be typically constrained by the relationship between the duration of immunity and CFR." This is beyond my understanding: smallpox could not really be constrained (or could not when it recurrently broke out in cities in the 18th century). SARS-CoV-2 was in contrast contained by human mitigation, and had little directly to do with CFR.*
- **Response: **We regret any confusion. In this study, we discuss both evolutionary constraints as well as the reduction in the rate of contact for symptomatic hosts, containment. We demonstrate that the evolution of smallpox-like viruses is likely constrained so that the CFR of smallpox remained high throughout the entire period over which it was endemic to human populations – well in line with the reviewer’s comments. We do not suggest that smallpox was historically contained; however, we do demonstrate that due to the relatively few asymptomatic transmission events associated with smallpox, in principle, containment may be possible for a modern society. Furthermore, we would argue that the CFR of SARS-CoV-2 played a key role in its containment. Should a novel virus have emerged with a much lower CFR, containment strategies would likely not have been pursued at all.

- *A minor point is the strange discussion of reversible chemical reactions in a paragraph on page 2. It seems to assume that energy is directly proportional to transmission rates. Also its relation to the rest of the manuscript is unclear.*
- **Response: **This discussion serves to introduce the concepts of kinetic and thermodynamic constraints to endemic equilibrium analogous to kinetic and thermodynamically limited chemical reactions. We believe that this analogy is illustrative of the evolutionary trajectories of viruses on multidimensional fitness landscapes and include this discussion in an effort to make the study more approachable to a broad audience. As is the case with Figure 1, we acknowledge that an expert reviewer may find this discussion unnecessary or perhaps even distracting. We apologize for any confusion and have further expanded the explanation of the idea of kinetic vs. thermodynamic constraints in the revised version in an effort to better connect this illustration to the topic at hand.

- *Overall, the paper presents a worthwhile investigation into an important phenomenon. However, it is very difficult to read and evaluate given its unclear assumptions (and complicated sentences). I would encourage a major rewrite, emphasizing which few (hopefully) parameters that are important, and  with new figures that more clearly illustrate the main point.*
- **Response: **We thank the reviewer for their comments. We have substantially revised and amended the text in an effort to clarify the presentation; however, we have elected to maintain the original figures.

# Peer review: Reviewer response for version 1

**DOI:** 10.5256/f1000research.56772.r87356

**Published:** 2021-06-25

**Authors:** Claus O. Wilke

**Affiliations:** 1 Department of Integrative Biology, The University of Texas, Austin, TX, USA

Overall, this contribution is very well written and seems reasonable. I have no major concerns, but I do have a few comments:

1. The authors write "as well as the difficulty of vaccination against lower-CFR viruses, such as influenza, relative to higher-CFR viruses, such as smallpox". I don't believe that difficulty of vaccination is directly linked to CFR, except maybe in the sense that there's less economic incentive to develop a vaccine if CFR is low.
2. Table 1 looks a bit weird. Maybe use two columns?
3. While the paper is overall well written, in the end I find it difficult to figure out what exactly the authors' conclusion is. I think the problem lies in the term "kinetically constrained", which is used throughout the manuscript but never actually explained. In the end, evolution of CFR comes down to the existence (or lack thereof) of transmission-mortality trade-offs, and I think you're trying to say that these trade-offs can be shaped by the rate constants of the various "reactions" in your model. If so, can you say this explicitly? And also, would this be at all surprising? Can you make any stronger or more specific statements?

You may find this paper helpful as a demonstration of how one can talk about this topic in very clear and understandable terms (Bull *et al. *(2014 ^1^)).

Is the work clearly and accurately presented and does it cite the current literature?

Yes

If applicable, is the statistical analysis and its interpretation appropriate?

Yes

Are all the source data underlying the results available to ensure full reproducibility?

No source data required

Is the study design appropriate and is the work technically sound?

Yes

Are the conclusions drawn adequately supported by the results?

Yes

Are sufficient details of methods and analysis provided to allow replication by others?

Yes

Reviewer Expertise:

mathematical modeling, evolutionary biology, infectious disease

I confirm that I have read this submission and believe that I have an appropriate level of expertise to confirm that it is of an acceptable scientific standard, however I have significant reservations, as outlined above.

## References

[1] : Theory and empiricism in virulence evolution.*PLoS Pathog*.2014;10(10) : 10.1371/journal.ppat.1004387e100438710.1371/journal.ppat.100438725340792PMC4207818

# Response

**Published:** 2021-07-17

**Authors:** Eugene Koonin

**Affiliations:** National Center for Biotechnology Information (NCBI), National Institutes of Health (NIH), USA

**Response to Claus Wilke:**

- *Overall, this contribution is very well written and seems reasonable. I have no major concerns, but I do have a few comments.*
- **Response: **We thank the reviewer for their kind remarks.
- *The authors write "as well as the difficulty of vaccination against lower-CFR viruses, such as influenza, relative to higher-CFR viruses, such as smallpox". I don't believe that difficulty of vaccination is directly linked to CFR, except maybe in the sense that there's less economic incentive to develop a vaccine if CFR is low.*
- **Response: **We agree with the reviewer that economic incentive is an important consideration which we did not mention in the previous version and have added in the revision. With this sentence, however, we intended to highlight the fact that lower CFR viruses are less likely to provide permanent immunity which in turn complicates vaccination strategies. This has been clarified in the revision.

- *Table 1 looks a bit weird. Maybe use two columns?*
- **Response: **We apologize if the former layout reduced the legibility of Table 1. We have reformatted the table as suggested.
- *While the paper is overall well written, in the end I find it difficult to figure out what exactly the authors' conclusion is. I think the problem lies in the term "kinetically constrained", which is used throughout the manuscript but never actually explained. In the end, evolution of CFR comes down to the existence (or lack thereof) of transmission-mortality trade-offs, and I think you're trying to say that these trade-offs can be shaped by the rate constants of the various "reactions" in your model. If so, can you say this explicitly? And also, would this be at all surprising? Can you make any stronger or more specific statements?*
- **Response: **We regret the confusion and have further expanded our discussion of kinetic vs. thermodynamic constraints in the revised version. Regarding the point that we are, “trying to say that these trade-offs can be shaped by the rate constants”, this is certainly the case, and we agree with the reviewer that this is to be expected. In this work, however, we highlight the opposite. There are very broad trade-offs (or in some instances the lack of trade-offs), which are largely independent of model parameters and shared across broad regimes within the life history characteristic phase space of all human respiratory viruses. We infer these very general evolutionary constraints without the use of an explicit trade-off function and without specifying an intra-host evolutionary model. This results in a “10,000 foot view” of the landscape, very general but not highly precise. In the original manuscript, we were cautious not to overstate the predictive value of our approach for specific epidemic outcomes. We thank the reviewer for this comment and acknowledge that these efforts may have resulted in unnecessary ambiguity in the text. We have strengthened and reiterated the statement of the principal conclusions throughout the revised version.
- *You may find this paper helpful as a demonstration of how one can talk about this topic in very clear and understandable terms (Bull et al. (2014)).*
- **Response: **We thank the reviewer for bringing this work to our attention. We agree that it is highly relevant,  most interesting and very clearly written. We have appropriately cited this work.
